# Immunotherapy for Breast Cancer Treatment

**DOI:** 10.52547/ibj.25.3.140

**Published:** 2021-03-08

**Authors:** Miganoosh Simonian, Mozhan Haji Ghaffari, Babak Negahdari

**Affiliations:** Department of Medical Biotechnology, School of Advanced Technologies in Medicine, Tehran University of Medical Sciences, Tehran, Iran

**Keywords:** Antibodies, Breast cancer, Immunotherapy

## Abstract

Breast cancer, as a heterogeneous disease, includes a wide range of pathological and clinical behaviors. Current treatment protocols, including radiotherapy, chemotherapy, and hormone replacement therapy, are mainly associated with poor response and high rate of recurrence. Therefore, more efforts are needed to develop alternative therapies for this type of cancer. Immunotherapy, as a novel strategy in cancer treatment, has a potential in treating breast cancer patients. Although breast cancer has long been considered problematic to treat with immunotherapy, as it is immunologically "cold," numerous newer preclinical and clinical reports now recommend that immunotherapy has the capability to treat breast cancer patients. In this review, we highlight the different immunotherapy strategies in breast cancer treatment.

## INTRODUCTION

Throughout the world, breast cancer is considered as one of the most prevalent cancer among women and the second most cancer worldwide^[^^1^^]^. Breast cancer is also more frequent in developed countries^[^^2^^]^, and annually, there is a 2% rise in its prevalence worldwide^[^^3^^]^. Such a rapid increase in the number of affected people has been reported from countries with the low incidence of breast cancer^[^^4^^]^. In the Middle East, this cancer is ranked in the first place. Similarly, in Iran, the incidence of breast cancer is estimated to be 23.1 per 100,000 women^[^^5^^]^, and the available data indicates that the disease prevalence has elevated in the country so that since 1999, it has stood in the first place among cancer cases in women nationwide^[^^6^^]^. In recent years, breast cancer is considered as a group of diseases, containing at least 21 distinct histological subtypes and four main molecular subtypes, which are constantly correlated with distinguishing clinical presentations and/or outcomes^[^^7^^,^^8^^]^. The most common type of invasive breast cancer (more than 75%) is now histologically specified as “no special type,” and called “ductal” carcinomas. Invasive lobular carcinoma is the most prevalent special histologic subtype accounts for about 15% of invasive breast cancers^[^^9^^]^. Table 1 summarizes the new classification of breast tumors reported by World Health Organization^[^^10^^]^. Breast cancer molecular subtypes are categorized through high-throughput microarray-based gene expression profiling. At the molecular level, there are four different molecular subtypes of breast cancer: luminal, normal breast-like, HER2, and basal-like. Luminal A (HR+/HER2-) is the most prevalent type of breast cancer that grows slower and tends to be less aggressive when compared with other subtypes. Luminal B (HR+/HER2+) is associated with poorer prognosis as it demonstrates a higher grade than luminal A (the higher proportion of breast cancer patients dropped to grade III and IV category in luminal B)^[^^11^^,^^12^^]^. Basal-like (HR-/HER2-) cancers are known as triple negative since they are ER-, PR- and HER2-. The incidence rate of TNBC is very high in black women and those with a *BRCA1* gene mutation. The worst prognosis can be found in this group of cancers^[^^13^^]^. In the last type of breast cancer, HER2-enriched (HR-/HER2+) patients have the chance of targeted therapies. This novel immunotherapeutic strategy brings favorable outcomes for these patients^[^^14^^,^^15^^]^.

**Table 1:** Histological typing of breast carcinomas^[^^165^^]^

Non-invasive lobular neoplasia
Lobular carcinoma *in situ* (classic, florid, and pleomorphic)
DCIS
DCIS of low nuclear grade
DCIS of intermediate nuclear grade
DCIS of high nuclear grade
Invasive breast carcinoma
Invasive breast carcinoma of no special type (including medullary pattern, invasive carcinoma with neuroendocrine differentiation, carcinoma with osteoclast-like stromal giant cells, pleomorphic pattern, choriocarcinomatous pattern, melanocytic pattern, oncocytic pattern, lipid-rich pattern, glycogen-rich clear cell pattern, and sebaceous pattern)
Microinvasive carcinoma
Invasive lobular carcinoma
Tubular carcinoma
Cribriform carcinoma
Mucinous carcinoma
Mucinous cystadenocarcinoma
Invasive micropapillary carcinoma
Carcinoma with apocrine differentiation
Metaplastic carcinoma (low-grade adenosquamous carcinoma, [high-grade adenosquamous carcinoma], fibromatosis-like metaplastic carcinoma, spindle cell carcinoma, squamous cell carcinoma, metaplastic carcinoma with heterologous mesenchymal [e.g. chondroid, osseous, rhabdomyoid, neuroglial) differentiation, and mixed metaplastic carcinomas)
Acinic cell carcinoma
Adenoid cystic carcinoma
Secretory carcinoma
Mucoepidermoid carcinoma
Polymorphous adenocarcinoma
Tall cell carcinoma with reversed polarity
Neuroendocrine neoplasms
Neuroendocrine tumor (grades 1 and 2)
Neuroendocrine carcinoma
Papillary neoplasms
Papillary ductal carcinoma *in situ*
Encapsulated papillary carcinoma
Solid papillary carcinoma (*in situ* and invasive)
Invasive papillary carcinoma
Epithelial-myoepithelial neoplasms
Malignant adenomyoepithelioma
Epithelial-myoepithelial carcinoma
Tumors of the male breast
*In situ* carcinoma
Invasive carcinoma

**Different mechanisms of immune evasion in breast tumors**

One of the major features of cancerous cell is its ability to escape and hide from adaptive immune responses^[^^16^^]^. Diverse mechanisms such as defective activation of tumor-directed T-cells, imperfect T-cell penetration into the tumor milieu, or emergence of resistance to immune cells action can participate in tumor evasion process^[^^17^^]^. Genomic instability, an evolving hallmark of breast cancer, resulted in the production of tumor neoantigens. Although these neoantigens can easily be distinguished by immune system and eradicated through T-cell function and immunity against tumor^[^^17^^,^^18^^]^, cells can demonstrate rather different immunogenic behaviors, conditional to different subtypes of breast cancer^[^^19^^]^. In the particular profiling study, suspicious calcifications are related to hampered immune system activity as well as ERBB2 overexpression^[^^20^^]^. Thus, breast calcifications could be beneficial to the management of patients with breast cancer for immunotherapy. Historically, these tumors are immunologically silent^[^^17^^]^ or “cold” , which means the attendance of low neoantigen burden and negligible effector tumor-infiltrating lymphocytes. Due to an obstacle to T-cell-based immunotherapies when confronting with non-inflamed tumors, several studies have attempted to discover new approaches to expand immune cell infiltration to tumor microenvironment and subsequent improvement of patient’s prognosis. Besides, direct tumor cell damage through the local tumor hyperthermia, serves as another valuable immunotherapy strategy for cancer, which has shown promising results in breast cancer patients^[^^21^^-^^23^^]^. Hyperthermia augments tumor cell sensitivity to antitumor immunological responses by boosting tumor surface HLA-I polypeptide-related sequence A expression. This specific sequence sensitizes tumor cell to natural killer cells and CD8^+^ cell-mediated lysis through the elevated levels of heat shock proteins and increasing exosomes release from tumor cells, respectively^[^^21^^]^. Recognition of tumor cells is another key step toward a successful immune response. In this context, tumor immune escape can take place in high levels of estrogen. Excessive estrogen may attenuate IFN-γ signaling and HLA-II expression, with apparent negative effect in all immune cells^[^^24^^]^. Moreover, estrogens enhance tumor cell survival and proliferation gene expression, along with growth factors (i.e. VEGF and EGF^[^^25^^]^). Since the presence of estrogen has beneficial effects on tumor development, antiestrogen therapies maybe a logical approach to improve the response to immunotherapeutic agents. On the other hand, estrogen deprivation initiates transcriptional events in favor of the tumor evasion and metastasis in patients receiving adjuvant hormonal therapy joints with HER2-targeted agents^[^^26^^]^. Therefore, blocking the PD-1/PD-L1 pathway in combination with hormone therapies should be applied with caution. Considering the reasons mentioned above, targeting growth factors by conventional mAbs has positive immunotherapy consequences by refining APCs activity^[^^27^^,^^28^^]^.

Resistance to mAb-based immunotherapies largely depends on possible pathways such as the activation of immuno-suppressive checkpoint pathways^[^^29^^]^. Although the blockade of the PD-1/PD-L1 pathway by FDA has been approved, atezolizumab appears to be among encouraging methods for immunotherapy. Indeed, it could achieve only 53% response rate for metastatic breast cancer versus 33% for the placebo group^[^^30^^]^. Similar to the PD-L1 pathway, PD-1 inhibitors have demonstrated the modest but promising results when administrated in breast cancer patients^[^^17^^,^^19^^]^. In this regard, PD-L1 status is considered the central point for anti-PD-1/PD-L1 therapies^[^^31^^]^; however, controversy remains regarding the prognostic value of PD-L1 expression^[^^17^^]^. Therefore, there is an urgent need to improve strategies for cancer immunotherapy as well as development and validation of novel biomarker panels.

Selection of apoptosis-resistant cells is another major hurdle, limiting immunotherapy success^[^^16^^]^. Since cellular apoptosis machinery can be activated by chemo- and immuno-therapies, tumor cell sensitivity to anticancer treatments can remarkably be influenced by the expression of anti-apoptotic factors^[^^32^^]^. Since different alternations in antiapoptotic proteins such as BCL-2, BMF^[^^32^^]^, and various pro-survival kinases^[^^33^^]^ were detected in patients with metastatic breast cancer, the justification of systematic classification techniques may be an inevitable approach in patients’ selection, in order to find the subjects with better response to immunotherapy and combined treatments of protein inhibitors. 

HLA-I surface expression has a great impact on success in T-cell-mediated immunotherapies. Thus, even small changes in the expression of HLA-I may confront breast cancer immunotherapy with a huge challenge^[^^16^^,^^34^^]^. HLA-I expression was lost in 70% of the lymph node metastases, 37% of *in situ* breast carcinomas and 43% of the primary tumors^[^^35^^]^. Further studies are required to determine those breast cancer patients obtained more benefit from immunotherapies. Variable levels of HLA-I expression was detected in triple-negative breast tumors^[^^36^^]^. Alternation in HLA-I expression is involved in immunosuppressive mechanisms and induces immune escape of tumor. Also, HLA-II presentation pathway activation leads to the infiltration of lymphocytes into tumor and improved prognosis^[^^37^^,^^38^^]^. For these reasons, before immunotherapy, specific considerations related to these receptors should be taken into account. It is also worth emphasizing that HLA-I expression may increase due to targeting mitogen-activated protein kinase or HER2^[^^39^^,^^40^^]^; hence, protein kinase inhibitors would be useful in the augmentation of T-cell-based immunotherapies.

**Rational for breast cancer immunotherapy**

**Fig. 1:**
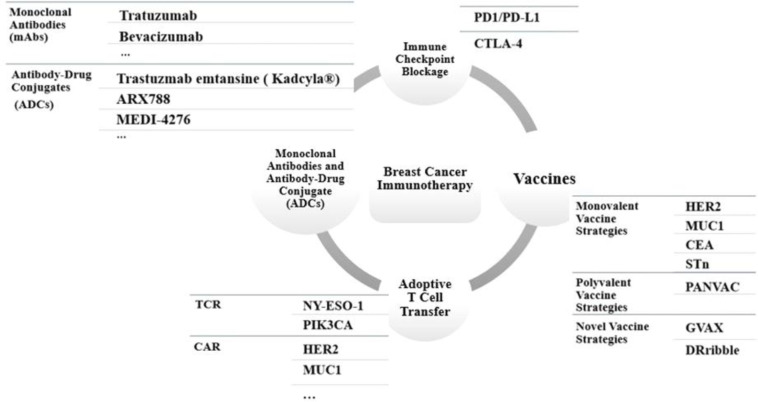
Four different immunotherapy strategies in breast cancer

Along with surgery as the main approach in the physical removal of the tumor, strategies such as radiation and chemotherapy can promote DNA damage or disrupt cell cycle, eventually leading to the death of cancer cells. No optimal chemotherapy regimen was identified for all subtypes of this heterogenous disease. Condensed chemotherapy appears to be more effective than conventional treatments; however, such chemotherapies require the growth factor usage, which substantially imposes additional costs to the patient. Additionally, the toxicity of chemotherapy impacts many organs. Vomiting, hair loss, chemotherapy-induced nausea, myeloid cell suppression, and neuropathy are the most frequent side effects^[^^41^^]^. Hormone therapy, like chemotherapy and radiation therapy, is a common non-targeted treatment and often causes severe side effects^[^^42^^]^. Owing to the limitations of traditional therapies, researches have focused on developing targeted therapies, giving rise to sustainable responses. Targeted treatment represents a great hope in the fight against cancer. Immunotherapy has recently been considered as a powerful treatment that targets a specific protein. Targeted therapy imposes a minor impact on normal cells and subsequently, low adverse effects^[^^43^^]^. Immunotherapies provoke the body's immune system to diagnose and eliminate the malignant cells. Various strategies include (1) immunological checkpoint inhibition, (2) anti-tumor vaccines, (3) transmission of elective T-cell treatment, and (4) immunotherapy using mAbs (Fig. 1).

**Immunological checkpoint inhibition**

Breast tumors show a high level of PD-1, PD-L1 expression, CTLA-4, and indoleamine-2,3 dioxygenase, all of which can improve anti-tumor immunity as treatment targets^[^^44^^]^. CTLA-4 is an encouraging therapeutic way to boost anti-breast cancer immunity. Human mAb against CTLA-4, ipilimumab, was approved in 2011 for the metastatic melanoma treatment. There have currently been some clinical trials assessing the immunity and effectiveness of CTLA-4 block in the breast cancer. In phase 1 clinical trial, the therapeutic effects of ipilimumab combined with nivolumab, an anti-PD-1 mAb and a histone deacetylase inhibitor, was investigated in HER2-negative breast cancer patients (The National Clinical Trial number (NCT) 02453620). In a separate study, ipilimumab in combination with nivolumab was assessed in the treatment of the early stages of breast cancer (NCT 02833233). The immunity of another anti-CTLA-4 mAb, tremelimumab, was evaluated as a single treatment or with anti-PD-L1 (durvalumab) in advanced breast cancer treatment (NCT02527434 and NCT01975831). Although CTLA-4 block is an attractive approach for treating the breast cancer, its immunity-related toxicity is still a matter of concern. Because the CTLA-4 limits the T-cells colony expansion and activation, its blocking would reduce the required threshold for T-cell activation; consequently, it would often be accompanied by autoimmune intense side effects and immunity-related ones, such as colitis, dermatitis, and hypophysitis ^[^^45^^]^. 

PD-1 is a cell surface receptor that binds to PD-L1 (B7-H1) or PD-L2 (B7-DC) and inhibit T-cell function in non-lymphoid and lymphoid organs^[46]^. The PD-1 expression is easily detectable in tumor-infiltrating lymphocytes and linked to poor prognosis^[47]^. PD-L1 has been found to be overexpressed in breast cancer cells and accompanied by poor prognosis, namely, the advanced tumor grade and increased proliferation rate^[^^48^^,^^49^^]^.

PD-1 and PD-L1 are currently being applied for therapeutic purposes. Pembrolizumab, an anti PD-1 human mAb, was tested in advanced TNBC patients (NCT01848834)^[^^50^^]^. Expression of PD-L1 was detected in 60% of patients. The pembrolizumab effect was observed in 27 cases; the overall response was 18.5%, and one patient was seen with a full response.

Atezolizumab is a human mAb that causes PD-L1 inhibition. It was examined with paclitaxel in 32 patients systematically treated for up to three years in the previous line^[^^51^^]^. Neutropenia was observed in 40% of cases, but no fatalities were reported; anti-tumor efficacy was found in 70% of TNBC patients. 

Avelumab is another completely humanized antibody that has been evaluated in a clinical trial of phase 2 in breast cancer patients^[^^52^^]^. Its ineffectiveness was observed in 40 out of 168 patients (23.8%). While these types of treatments have acceptable and promising results, it should not be neglected that in some cases, they can result in drastic side effects, e.g., death in several patients. Further studies are vital to assess the risk of this type of treatment. On the other hand, it seems that immunosuppressive mechanisms among various patients are different, which leads to exorbitant costs for each one. Moreover, this treatment could have adverse effects by systematic suppression of the immune system.

**Antitumor vaccines**

The aim of antitumor therapy vaccines is to attain an extremely specific antitumor cellular immune response. The effectiveness of antitumor vaccines is mainly rely on the stimulation of tumor-specific T lymphocytes to detect and eradicate the proliferated cancer cells^[^^53^^]^. In addition, early T-cell responses could prevent tumor recurrence by inducing long-term immune memory^[^^54^^]^. Identifying mutated tumor antigens could assist the expansion of personal vaccination strategy. Several vaccine approaches, comprising of monovalent, polyvalent, and cellular vaccines, have been assessed. Monovalent vaccines were designed utilizing single TAA (such as HER2, sialyl-Tn, carcinoembryonic antigen, mucin 1, Wilms tumor gene, and telomerase reverse transcriptase) to stimulate an inherent antitumor response to aid cancer treatment^[^^55^^]^. Although appeared to be efficacious in targeting immune responses toward the specific antigen, monovalent vaccines may simplify the appearance of resistant tumor cells with decreased expression of tumor antigen^[^^56^^]^. To alleviate the negative outcomes of antigen loss, polyvalent vaccines have been developed. These vaccines use multiple TAAs to enable more drastic and varied antitumor responses. One example is PANVAC (pancreatic vaccine), a recombinant poxvirus-vector therapeutic vaccine that stimulates immune responses against the tumor antigens (carcinoembryonic antigen and MUC1). In phase 2 clinical study, more encouraging effects were detected in the vaccination group compared with the chemotherapy-alone group (69% vs. 53%)^[^^57^^]^. However, polyvalent vaccines could carry multiple TAAs simultaneously. The third class of vaccines aims to improve the delivery of TAAs through the whole cell manufacture or cellular contents. GVAX, a granulocyte-macrophage colony-stimulating factor gene-transfected tumor vaccine^[^^58^^]^. A phase 2 study was performed with GVAX in combination with cyclophosphamide and trastuzumab in HER2-negative metastatic breast cancer (NCT00971737). Anti-tumor vaccines are recognized as an effective strategy for treating breast cancer and indicate spectacular features with acceptable toxicity profiles. However, there are limitations for these vaccines, namely, the imperfect antitumor immune response which is inadequate for tumor elimination^[^^59^^,^^60^^]^. The downsides of major histocompatibility complex in peptide vaccines are that each vaccine is used in a group of patients with expression of specific HLA molecules^[^^61^^]^. Systemically, injected peptides bind to non-professional APCs, which could lead to tolerance because of inefficient stimulation. In addition, the effectiveness of the vaccine is greatly affected by the very short half-life of peptides in the body^[^^62^^]^. 

Overall, it is necessary to identify a set of antigens associated with tumor in each individual to optimize the production of stronger and more specific anticancer cells; for instance, vaccines. Targeting multiple antigens in these strategies is crucial not only to prevent the loss of the antigen due to selective pressure but also to enhance the chances of inducing immune memory that prevents metastasis and recurrence of the tumor. Eventually, although antitumor vaccines would bring immunity for breast cancer patients, their effectiveness is less than expected.

**Adoptive T-cell transfer therapy**

Transmission of selected T cells contains the extraction of patient T cells and also the genetic or chemical alteration to increase their activity, with the aim of creating antitumor immunity^[^^63^^]^. It has been observed that in a single tumor sample, there is a different population of lymphocytes with various antigenic characteristics and phenotypic populations^[^^64^^]^. Extraction and manipulations to stimulate tumor-infiltrated lymphocytes did not indicate positive clinical effects in immunotherapy of breast cancer^[^^65^^]^. Improvements in molecular biology and genetic engineering have resulted in the development of two new types of ACTs: (1) TCR gene transfer and (2) CAR gene transfer.

**TCR**

 Gene transfer technology helps to develop new strategies in ACT category. Effector lymphocytes are made by the fusion of the patient's T cells with genes that encodes antigen receptors. These cells have the capability of eliminating tumor *in vivo*^[^^66^^]^. The T-cells redirection is achieved by expressing an antigen-specific TCR on cell surface, providing a recognition signal for T cells, followed by a co-stimulatory signal to regulate the T cells activation against tumors. TCRs may have better function to transform cancer therapeutics compared to tumor**-**infiltrating lymphocyte-strategies since they have enhanced tumor specificity and the possibility of clinically relevant doses of the production of these therapeutic cells. These approaches could generate more efficient T cells for targeting tumors without requirement for a new T-cell activation overcoming the central and peripheral tolerance fundamental limitations^[^^67^^]^. A TCR may target either intracellular (it could add to the pool of potential targets) or extracellular antigen in the context of significant major histocompatibility complex presentation^[^^68^^]^. A cancer testis antigen, NY-ESO-1, is expressed in 10% of TNBCs and has been revealed to be effectively targeted with TCR transfer immuno-therapy^[^^69^^]^. Targeting PIK3CA, a common driver oncogene using genetically redirected T cells, eradicated target cancer cells^[^^70^^]^. 

**CAR**

CAR T cells consists of an antibody-binding domain and a cell signaling domain. Elements that enhance T-cell persistence and activity are also included in CAR constructs^[^^71^^]^. CARs specific for a broad range of antigens have been developed and effective treatment of breast cancer with these reported in several in vivo studies. Different CAR T-cells targeting, mucin 1, folate receptors and HER2 -MUC1 are available ^[^^72^^-^^74^^]^ . Adoptive cell therapy is a personal-centered therapy and the choice of different approaches and is strongly influenced by each patient’s condition. Such treatments require complex preparations and procedures for each patient, long-term cell culture, experts, and patient preparation^[^^75^^]^. Moreover, immune cells from cancer patients show different phenotypes when compared to healthy donors. One of the challenges in using patient’s leukocytes is that the T cells and dendritic cells obtained from cancer patients cannot function properly in many cases. Additionally, whether they can create a desired anti-tumor immunity or not is of concern^[^^76^^]^.

**Adoptive immunotherapy with mAbs and ADCs**

mAbs have been developed over the past two decades. Trastuzumab was the first mAb targeting the extracellular HER2 cell domain, leading to the cessation of mitogen-activated protein kinase and PI3K/AKT intracellular signaling in the *in vivo* and *in vitro* environment. This antibody was finally received approval from FDA in 1998^[^^77^^,^^78^^]^. Unfortunately, only one-third of patients with HER2 amplification respond to this treatment^[^^79^^]^. Genetic changes, such as decreased expression or mutations in HER2^[^^80^^]^, and the PIK3CA downstream mutation or PTEN loss of function, could affect the response to the anti-HER2 agent^[^^81^^,^^82^^]^. Bevacizumab was another mAb that could regulate angiogenesis and tumor survival against VEGF^[^^83^^]^. However, angiogenesis inhibitors have not been successful in treating breast cancer. Failure to target angiogenesis is one of the most significant experiments in late 2000 clearly showing that angiogenesis is not a central mechanism for the disseminated tumor cells or micrometastatic disease.

Pertuzumab is a type of human monoclonal IgG antibody and has therapeutic confirmation in combination with trastuzumab for HER2-positive metastatic breast cancer patients^[^^84^^]^. Meanwhile, mAbs activate the immune response to kill cancer cells. For instance, opsonized epithelial tumor cells by trastuzumab could be killed through antibody-dependent cytotoxicity via natural killer cell^[^^85^^,^^86^^]^. However, *de novo* mutations occur in 65% of patients, and 70% of the patients who initially respond to treatment eventually become resistant^[^^87^^]^.

The tyrosine kinase receptor family EGFR plays an important role in tumor formation. Evidence has shown that prescribing two inhibitors against EGFR or ErbB2 family works in perfect harmony and significantly increases antitumor activity. For instance, combination therapy with trastuzumab and lapatinib, an EGFR/ HER2 dual inhibitor, results in perfect cessation of BT474 breast cancer cells. These studies have also been extended to HER2-positive MCF7 breast cancer model^[^^88^^]^.

PI3K/AKT/mTOR is a key pathway in breast cancer. Everolimus and Palbociclib are proven anti-mTOR and anti-CDK4/6, respectively, which can be used to treat both ER-positive and HER2-negative breast cancer^[^^89^^]^. However, everolimus treatment can cause negative feedback to IRS-1/IRS-2, thus activating mTORC2 and AKT downstream signals^[^^90^^]^. Furthermore, the toxicity of the PI3K pathway inhibitor and the reduction of total survival are needed to be considered^[^^91^^]^. Regrettably, no targeted treatment other than standard chemotherapy for triple-negative breast cancer is recommended. This type of breast cancer is extremely invasive and requires continuous angiogenesis at all stages of tumor growth and expansion. Anti-vascular endothelial growth factor mAbs, bevacizumab, were confirmed by FDA; however, it was excluded due to its limited effect on the general survival of patients^[^^92^^,^^93^^]^. None of the anti-VEGF antibodies, ramucirumab, tyrosine kinase inhibitors, sunitinib, and sorafenib are effective in improving the survival of TNBC patient in phase 3 of clinical trial^[^^94^^,^^95^^]^. In addition, disappointing results from EGFR and cetuximab antibodies have been reported in clinical trials for TNBC^[^^96^^,^^97^^]^.

In the majority of cases, a specific mutation or a signaling pathway is targeted. Unfortunately these signal transmission pathway inhibitors are moderately efficient. Evidence has suggested a very low survival rate in several weeks or months, if metastatic condition is present, which is due to the significant limitations of current targeted therapies^[^^98^^]^. Reasons for internal resistance to targeted drugs include temporary antitumor activity, lack of attention to heterogeneity among patients, and heterogeneity in the tumor itself, as well as the lack of comprehensive insights into how cancer genomes and molecular networks regulate gene expression^[^^98^^,^^99^^]^. Moreover, large molecules such as mAbs have a poor distribution due to their high molecular weight, which this could be an explanation for the difficult treatment of large solid cancerous tumors by mAbs^[^^100^^]^. To overcome these challenges, targeted therapies have emerged in the form of antibody-drug conjugates. Cytotoxic drugs are supposed to bind to antibodies via chemical linkers. The carrier antibody could detect cancerous antigen cells and deliver the conjugated drug to those cells^[^^101^^,^^102^^]^. ADCs are assumed perfect delivery systems for cytotoxic antitumor drugs^[^^103^^]^. Key factors for ADC development include the selection of the target antigen and the features of linker between the antibody complex and the drug. Other important factors are comprised of drug to antibody ratio and the effects of drug conjugation on antibody function. The first FDA approved ADC was in 2001 for acute myeloid leukemia patients. Gemtuzumab ozogamicin with the brand name of Mylotarg® was removed from the market in June 2010. In 2017, the product was re-introduced to the US market^[^^104^^]^. The second and third ADCs offered to the commercial market were Brentuximab vedotin with the brand name of Adcetris® and Trastuzumab emtansine with the brand name of Kadcyla. Brentuximab vedotin was approved for patients with Hodgkin's lymphoma on August 19, 2011^[^^105^^]^, while Trastuzumab emtansine was affirmed for metastatic breast cancer patients in February 2013^[^^106^^]^. The newest ADC, Inotuzumab ozogamicin, with the trade name of Besponsa®, entered the market by European Commission for the treatment of adults with lung, acute lymphoblastic leukemia. Inotuzumab ozogamicin was later confirmed on August 17, 2017, for treating adults with lung, acute lymphoblastic leukemia or resistance to acute lymphoblastic leukemia^[^^107^^]^. As breast cancer will remain a global public health problem for women in the future^[^^2^^]^, ADC could expand new methods and techniques in efficient treatments of breast cancer. Among 15 ADCs currently being evaluated for breast cancer, seven cases target the HER2. The HER2 recipient is one of the four receptors from the EGFR family and a protein receptor consisting of one extracellular domain and one intracellular domain. ADCs can be exceptionally effective in breast cancer with HER2 expression^[^^108^^]^. HER2 expression in tumor differs from normal tissue and increases in 20-25% breast cancer cases^[^^109^^]^. To date, Kadcyla® is the only ADC approved for HER2-positive metastatic breast cancer that has previously been treated with trastuzumab and taxane. T-DMI is composed of trastuzumab (humanized IgG1), conjugated with DM1, via an SMCC linker. T-DM1 has an average drug-antibody ratio of 3.5^[^^110^^,^^111^^]^. In these studies, T-DMI, an active drug with high tolerability, has shown a strong antigen activity in laboratory conditions, indicating an acceptable pharmacokinetic profile in xenograft samples of human tumor^[^^111^^]^. T-DMI active catabolite (Lys-SMCC-DM1) demonstrates strong activity after antigen-dependent entry and antibody lysosomal degradation. However, Lys-SMCC-DM1 has poor membrane permeability, explaining why it has the least bystander properties. In addition, 50% of metastatic breast cancer patients do not reply to this treatment^[^^112^^]^. Similarly, there are significant demands for the presentation of newer conjugates for HER2-positive tumors and other types of breast cancer.

There are six HER2-based ADCs in clinical trials aimed to improve the activity and maintain or enhance the T-DM1 immunity. Moreover, the utilization of the following cytotoxic drugs with different actions has been assessed: DS-8201a for the transmission of exatecan, a topoisomerase inhibitor^[^^113^^-^^116^^]^, and SYD985^[^^117^^,^^118^^]^, a DNA-alkylating drug^[^^119^^,^^120^^]^, as well as ADCT-502 for the transmission of pyrrolobenzodiazepine that enters DNA minor grooves^[^^121^^]^.

**ARX788**

ARX788 is an ADC with site specifically conjugated drug, an inseparable linker, and a combination of a linker and a cytotoxic drug that has amberstatin. Amberstatin (AS269) contains monomethyl auristatin F linked to a short polyethylene glycol spacer^[^^122^^]^. Preclinical ARX788 studies have shown activity in different xenograft models, such as ovarian and trastuzumab-resistant breast cancer.

**MEDI-4276**

Targeting HER2 can be carried out by both targeting different epitopes in HER2, as well as utilizing other cytotoxic drugs^[^^123^^,^^124^^]^. MEDI-4276 is completely a human IgG connected to a different epitope of HER2. This ADC is in the phase 1 of clinical trial in breast and gastric cancer patients.

**XMT-1522**

XMT-1522 uses a polystyrene-based polymer (Fleximer®) that significantly increases the loading of the cytotoxic drug on the antibody (DAR 12-15)^[^^125^^]^. The used mAb, XMT-1519, is attached to HER2 epitope different from those targeted by trastuzumab The drug used in XMT-1519 is a new auristatin having a unique medicinal property. 

**DS8201-a**

DS8201 is an anti-HER2 human mAb conjugated with topoisomerase I inhibitor, DXd. This ADC shows a cell-related HER2 toxicity effect in laboratory conditions in pancreatic, breast, and gastric cancer cells. Human model studies have also indicated HER2-specific activity in tumors with heterogeneous expression of HER2^[^^114^^,^^115^^]^. This ADC has entered in phase 1 of clinical trial. SYD985 contains conjugated trastuzumab connected to duocarmycin through maleimide coupling to inter-chain disulfides. The main difference in this ADC, in comparison with TDM-I, is the use of a degradable linker and DNA destructive agent. Duocarmycins are strong DNA alkylating agents that bind to the DNA groove and cause adenine N3 alkylation^[^^117^^]^. Peptide degradable linkers have good systemic stability. Proteolytic release occurs via cathepsin B and L, creating a self-decomposing short-lived intermediate. This ADC has entered phase 1 of clinical trial in solid cancers, which then enters the expansion phase in HER2-positive tumors. 

ADCT-502 is an ADC with engineered trastuzumab attached to highly cytotoxic PBD-based linker-drug tesirine. ADCT-502 is currently being evaluated in patients with solid tumors expressing HER2 in phase 1 of clinical trial. Other ADCs, with different targets and in the final stages of clinical trials are as follows:

**Glembatumumab vedotin**

GPNMB is a transmembrane glycoprotein that its role in cancer is complicated; It acts like a tumor suppressor or has a function in the cancer progression. In cancer, GPNMB overexpression is found in different types of tumors, including melanoma, breast, lung, and osteosarcoma, when compared to normal tissues^[^^126^^-^^129^^]^. In breast cancer, GPNMB gene expression is associated with reduced overall survival. GPNMB overexpression is observed in both TNBC and basal cancers, which is associated with poor prognosis^[^^130^^]^. 

Glembatumumab vedotin (CDX-011) is an anti-GPNMB ADC containing an IgG2 connected to a microtubule inhibitor, MMAE^[^^131^^]^, through a vc linker^[^^132^^,^^133^^]^. In a study of phase 2 clinical trials in patients with advanced cancer or local metastasis of breast cancer, Glembatumumab vedotin had more acceptable results when comparing to chemotherapy. Additionally, it had fewer side effects in patients with less stimulation of bleeding, itching, neuropathy, and alopecia^[^^134^^]^. 

**IMMU-132**

The TROP-2 is a membrane glycoprotein overexpressed in a variety of tumors, including breast cancers. Excessive expression of TROP-2 is observed in invasive disease, linked with drug resistance and poor prognosis^[^^135^^-^^137^^]^. IMMU-132 (Sacituzumab govitecan) is an anti-TROP-2 ADC consisting of one IgG1 anti-TROP-2 mAb (hRS7) and one topoisomerase inhibitor. Unlike other ADCs, SN-38, is a topoisomerase I inhibitor having a moderate effect (nM) when comparing to other drugs currently used in ADC (<200 pM). Second, its releasing mechanism is dependent on acid, which exerts through a benzyl carbonate bond to SN-38’s lactone ring. This linker contains a short sequence of PEG and a lysine residual, making it relatively polar in nature. It would likely explain the high level of 7.6 DAR in this ADC, which is twice the current ADCs. In PH near 5.3 of lysozyme (37 centigrade), 50% of the drug is released in 13 hours. IMMU- 132 preclinical models have shown specific antigen activity against TROP- 2-expressing cells in different *in vitro* and *in vivo*^[^^136^^,^^138^^]^. 

**SAR566658**

CA6 is a tumor-related antigen and one sialoglycotope of MUC1. It is thought that it results from inappropriate MUC1 glycolysis^[^^139^^]^. The CA6 cancer-related glycotype is observed in most normal tissues at low levels, whereas its overexpression has been found in many solid tumors, including 30% of breast cancer cases^[^^139^^,^^140^^]^. SAR566658 contains an anti-CA6 (huDS6) antibody that binds to a non-polar S-methyl-DM4 drug through a stable disulfide bond^[^^141^^]^. Two methyl groups close to disulfide in these linkers are to prevent the breakdown of the linker by free thiols in the bloodstream, while allowing the breakdown in the existence of a much higher level of glutathione and cysteine inside the cytosol or nucleus^[^^142^^]^. This ADC has indicated an acceptable immune and antitumor activity during phase 1 of clinical trial in acceleration dose of patient’s different solid tumors with CA6 (more than 30% of tumor cells) expression^[^^143^^]^. One phase 2 clinical trial is being conducted in TNBC patients with CA6 expression (NCT 02984683). Numerous ADCs are involved in phase 1 trials targeting breast tumor-related antigens.

**LIV-1**

LIV-1 (SLC39A6) is a transmembrane protein that transports zinc into cells^[^^144^^]^. LIV-1 regulates estrogen in breast cancer, which its expression has been linked to tumor development and metastasis^[^^145^^,^^146^^]^. LIV-1 expression is associated with E-cadherin decrease and may play a role in epithelial-mesenchymal transmission and increased metastasis^[^^147^^,^^148^^]^. The anti-mLIV2 mouse mAb precisely binds to an extracellular N-terminus epitope of LIV-1. Complementarity-determining region grafting was used to produce anti-LIV-1 human IgG1, named hLIV22. ADC guided to the (SGN-LIV1A) LIV1 side is produced by hLIV22 mAb connection to a MMAE (an analog auristatin) via endogenous cysteine. SGN-LIV1A leads to the elimination of ER- and LIV1-positive MCF7 breast cancer cells, as well as BR0555, which is a xenograft tumor of the breast cell^[^^149^^]^. SGN-LIV1A alone and in combination with trastuzumab are currently undergoing phase 1 clinical trial in patients with metastatic breast cancer expressing LIV1.

**PTK7**

PTK7 is identified as the colon carcinoma kinase 4, a highly protected PTK that plays an important role in Wnt signaling. In breast cancer, PTK7 is more expressed in ER-negative tumors than in ER-positive tumors. Moreover, PTK7 suppression through siRNA leads to severe inhibition of human ER-negative breast cancer growth^[^^150^^]^. An anti-PTK ADC with average DAR of 4, alongside a conjugated h6M24, human (IgG1) mAab, is connected to Aur0101, an auristatin analog, using a decomposable linker (vc-PABC). This ADC is called h6 M24-vc-0101 or PF-06647020. Aur0101 is specifically designed to maintain cellular potential and oxidative metabolism faster than MMAE^[^^151^^]^. Increased clearance of this cytotoxic drug may reduce systemic toxicity and increase ADC therapeutic indicators. PF-06647020 produces antigen-dependent cytotoxicity in PTK7-expressive cells, leading to the cessation of cell mitosis and destruction of the microtubule. Studies in TNBC models of NOD/SCID rats have shown a high anti-tumor activity of this ADC. Preclinical anti-tumor activity of acceptable immunity and PF-06647020 pharmacokinetic profiles lead to the entrance of ADC to the first phase of clinical trial studies in patients with advanced solid tumors and different expressions of PTK7. Later, it entered to cohort studies in TNBC, non-small-cell lung cancer, and ovarian cancer patients^[^^152^^]^.

**LAMP-1**

LAMP-1 (CD107a) and LAMP-2 are transmembrane type I proteins, accounting for about 50% of all lysosomal membrane proteins^[^^153^^]^. In normal cells, LAMP-1 is normally expressed in lysosomes, though it is transferred to the surface of tumor cells, where its expression level is associated with invasion and metastasis of different types of tumors^[^^154^^]^. SAR428926 is a LAMP1 ADC in which Ab-1 is connected to the LAMP1 luminal domain. This antibody does not detect LAMP1 in normal tissue cells. SAR428926 connects to DM4 using a decomposable link, N-succinimidyl-4-(2-pyridyldithio) butanoate. SAR428926 assessment of subcutaneous patient-derived xenograft mouse models indicate increased antigen-dependent anti-tumor activity, including shrinkage of breast, prostate, colorectal, lung, and ovarian tumors. The reported activity is related to the LAMP-1 level of expression and the tumor model sensitivity to the DM4^[^^155^^,^^156^^]^. P-cadherin has a major role in calcium-dependent cell-cell adhesion. It is expressed during growth and also in normal myoepithelial/basal cells of adults and epithelial tumors. In a normal breast, P-cadherin is involved in maintaining the breast epithelium structural integrity in the adult tissues of basal layer and hair follicles^[^^157^^-^^159^^]^. In breast cancer, P-cadherin overexpression is related to the invasive power of tumors and is a hallmark of poor prognosis^[^^129^^]^. PCA062 is an anti-P-cadherin ADC in which an IgG1 is connected to a maytansinoid DMI. PCA 062 is rapidly internalized inside the cell and then lysosome, leading to antigen-dependent cell toxicity. PCA062 ADC has anti-tumor activity in breast and bladder cancer xenograft models^[^^160^^]^. At present, PCA062 is evaluated in phase 1 clinical trial in TNBC patients with P-cadherin expression.

**EphA4**

Ephrin receptors, from RTK family, have an increased expression in tumors and are related to the development of different types of tumors, including breast, pancreatic and lung cancer^[^^161^^,^^162^^]^. Expression profiles of PDX models show an increase in the EphA4 expression of TNBC tumors, as compared with adjacent natural breast tissue and other breast cancer subtypes^[^^163^^]^. PF-06647263 is an anti-EphA4 ADC in which calicheamicin, DNA destructive agent, binds to an IgG1, anti-EphA4 mAb. It has been introduced to the market as Mylotarg® (Gemtuzumab ozogamicin)^[^^164^^]^. 

**Conclusions and future directions**

Immunotherapy has shown great potential for breast cancer treatment, demonstrating the possibility of utilizing the immune system for clinical benefit in this malignancy. The developments in targeted immunotherapy have led to clinical advances in the treatment of breast tumors. In near-term future, the advances in combination immunotherapies can alter breast cancers from immunologically cold tumors to immune-activated lesions ready for response to immunotherapy. Several strategies that utilize molecular targeted agents to boost breast cancer-specific immunity are under rapid development. In addition, combinatorial approaches that act on the compensatory pathways in resistant lesions may markedly raise hope on the effectiveness and duration of response to immune-based breast cancer prevention.

## CONFLICT OF INTEREST

None declared.
